# Oxidative stress augments toll-like receptor 8 mediated neutrophilic responses in healthy subjects

**DOI:** 10.1186/1465-9921-10-50

**Published:** 2009-06-15

**Authors:** Satoru Yanagisawa, Akira Koarai, Hisatoshi Sugiura, Tomohiro Ichikawa, Masae Kanda, Rie Tanaka, Keiichiro Akamatsu, Tsunahiko Hirano, Kazuto Matsunaga, Yoshiaki Minakata, Masakazu Ichinose

**Affiliations:** 1 Third Department of Internal Medicine, Wakayama Medical University, School of Medicine, Wakayama, Japan

## Abstract

**Background:**

Excessive oxidative stress has been reported to be generated in inflamed tissues and contribute to the pathogenesis of inflammatory lung diseases, exacerbations of which induced by viral infections are associated with toll-like receptor (TLR) activation. Among these receptors, TLR8 has been reported as a key receptor that recognizes single-strand RNA virus. However, it remains unknown whether TLR8 signaling is potentiated by oxidative stress. The aim of this study is to examine whether oxidative stress modulates TLR8 signaling in vitro.

**Methods:**

Human peripheral blood neutrophils were obtained from healthy non-smokers and stimulated with TLR 7/8 agonist imidazoquinoline resiquimod (R848) in the presence or absence of hydrogen peroxide (H_2_O_2_). Neutrophilic responses including cytokine release, superoxide production and chemotaxis were examined, and the signal transduction was also analyzed.

**Results:**

Activation of TLR8, but not TLR7, augmented IL-8 release. The R848-augmented IL-8 release was significantly potentiated by pretreatment with H_2_O_2 _(p < 0.01), and N-acetyl-L-cysteine reversed this potentiation. The combination of H_2_O_2 _and R848 significantly potentiated NF-kB phosphorylation and IkBα degradation. The H_2_O_2_-potentiated IL-8 release was suppressed by MG-132, a proteosome inhibitor, and by dexamethasone. The expressions of TLR8, myeloid differentiation primary response gene 88 (MyD88), and tumor necrosis factor receptor-associated factor 6 (TRAF6) were not affected by H_2_O_2_.

**Conclusion:**

TLR8-mediated neutrophilic responses were markedly potentiated by oxidative stress, and the potentiation was mediated by enhanced NF-kB activation. These results suggest that oxidative stress might potentiate the neutrophilic inflammation during viral infection.

## Introduction

Reactive oxygen species (ROS) such as hydrogen peroxide (H_2_O_2_) and superoxide anion are generated in inflamed tissues and are reported to contribute to the pathogenesis of inflammatory lung diseases including chronic obstructive pulmonary diseases (COPD) [1,2], bronchial asthma [3,4], cystic fibrosis [5,6], and idiopathic pulmonary fibrosis [7,8]. Large amounts of ROS derived from inflammatory cells cause pro-inflammatory cytokine production. In fact, H_2_O_2 _has been reported to augment cytokine production in previous studies [9,10]. Among inflammatory cells, neutrophils are a key player in the inflammatory lung diseases. It is well-known that excessive infiltration of neutrophils is observed in the airways during exacerbations induced by viral infections [11-14].

Toll-like receptors (TLRs) are simple pattern recognition receptor systems and are known to react with conserved molecular patterns of pathogens [15]. The innate immunity cells also act against viral infections through TLRs including TLR3, TLR7 and TLR8. Human neutrophils possess all functional TLRs except TLR3 [16], and their agonists enhance neutrophil functions such as cytokine release, superoxide generation and phagocytosis [16]. TLR7 and TLR8, located in the endosome, act as anti-viral receptors for recognizing single strand RNA (ssRNA) [17-19], which is present at various phases of viral infection from viral entry to replication. After TLR7 and TLR8 are activated by ssRNA, their signals are transduced through myeloid differentiation primary response gene 88 (MyD-88) and tumor necrosis factor (TNF) receptor-associated factor 6 (TRAF6) leading to enhanced nuclear factor-kappa B (NF-kB) DNA binding activity [20]. Activation of NF-kB leads to increased inflammatory gene products such as interleukin-8 (IL-8) and GM-CSF causing neutrophilic inflammation during viral infection. Resiquimod (R848), a potent synthetic agonist of TLR 7/8 has been reported to simulate the effects of ssRNA viruses on TLR 7/8, to prime human neutrophils [16,21], and then increase the biosynthesis of lipid mediators through NF-kB activation [22] suggesting that TLR7 and TLR8 activation might affect the neutrophilic responses.

Although excessive oxidative stress occurs in the airways of inflammatory lung diseases during exacerbations, it remains unclear whether oxidative stress potentiates the neutrophilic responses against viral infection. Therefore, by using human peripheral neutrophils from healthy never-smoking subjects, the present study was designed to clarify whether oxidative stress can potentiate the TLR8-mediated neutrophilic responses, including cytokine production, chemotaxis and superoxide generation. Furthermore, we also investigated what signal transductions are associated with this potentiation of the neutrophilic responses.

## Materials and methods

### Reagents

Commercially available reagents were obtained as follows: Mono-Poly Resolving Medium was from Dainippon Pharmaceutical Co. Ltd. (Osaka, Japan); fetal calf serum (FCS) and RPMI medium 1640 (RPMI 1640) were from Invitrogen (Carlsbad, California, USA); R848 (resiquimod: 4-amino-2-etoxymethyl-α,α-dimethyl-1*H*-imidazo [4,5-*c*]quinolin-1-ethanol), bafilomycin and 12-o-tetradecanoylphorbol 13-acetate were from Alexis Biochemicals (San Diego, California, USA); R837 (Imiquimod: 1-isobutyl-1*H*-imidazo [4,5-*c*]quinolin-4-amine) was from Biomol (Plymouth Meeting, Pennsylvania, USA); N-acethyl-L-cysteine, MG-132, dexamethasone and anti-β-actin antibody were from Sigma (St. Louis, Missouri, USA); anti-TLR8 rabbit polyclonal antibody was from Abgent (San Diego, California, USA); Cellfix solution was from Becton Dickinson (San Jose, California, USA); phycoerythrin (PE)- conjugated anti-TLR8 antibody solution was from Imgenex (San Diego, California, USA); dihydro-rhodamine-123 (DHR-123) was from Cayman Chemical (Ann Arbor, Michigan, USA); human recombinant IL-8 was from Acris antibodies (Hiddenhausen, Germany); anti-human MyD88 antibody, anti-human TRAF6, and anti-human IkBα were from Santa Cruz (San Diego, California, USA); peroxidase-conjugated secondary antibodies were from Rockland Immunochemicals (Gilbertsville, Pennsylvania, USA)

### Isolation of peripheral blood neutrophils

Healthy subjects participated in the present study. They were never-smokers and had had no infection for 4 weeks preceding the study. Human peripheral blood neutrophils were isolated from whole blood by a density gradient technique using Mono-Poly Resolving Medium as previously reported [23]. Briefly, whole blood was collected by vein puncture into tubes containing EDTA anticoagulant. Then, each blood sample was gently mounted onto the same volume of Mono-Poly Resolving Medium without mixing. The samples were centrifuged at 400 × *g *for 20 min at room temperature. The blood was separated into four layers from the top, plasma, lymphocytes/mononuclear cells, neutrophils, and red blood cells. The neutrophil layer was gently collected by a pasteur pipette without aspirating the other layers and put into fresh 20 ml tubes. This procedure allowed us to obtain neutrophils with over 95% purity and viability as determined by trypan blue staining. After washing by phosphate-buffered saline (PBS) solution and counting the cell numbers, neutrophils were suspended in 10% FCS in RPMI 1640 at a concentration of 1 × 10^6 ^cells/ml. The neutrophils were isolated before each experiment and used immediately. All replicate experiments in the current study were performed by using neutrophils from different donors. This study was approved by the local ethics committee of Wakayama Medical University School of Medicine. Informed written consent was obtained from all subjects.

### Immunocytochemistory

100 μl of the neutrophil suspension containing 1 × 10^5 ^cells were centrifuged by a Cytospin 4 cytocentrifuge (ThermoShandon, ThermoBioAnalysis, Tokyo, Japan) at 25 × g for 5 min. The preparation was fixed in 4% paraformaldehyde fixative solution for 30 min. Endogeneous peroxidase activity was blocked by incubation in 0.3% H_2_O_2 _in PBS for 15 min at room temperature. After washing, the cells were incubated with anti-TLR8 rabbit polyclonal antibody (1:100 dilution) for 12 hrs at 4°C. Non-specific binding to the antibody was prevented by pre-incubation with 2% bovine serum albumin in PBS containing 0.3% Triton-X for 30 min. The immunoreactions were visualized by the indirect immunoperoxidase method using Envision polymer reagent, which is goat anti-rabbit IgG conjugated with peroxidase labeled dextran (Dako Japan Ltd, Kyoto, Japan), for 1 hour at room temperature. Diaminobenzidine reaction was performed, followed by counterstaining with hematoxirin. The slides were viewed with a microscope (BX-50, Olympus Corporation, Tokyo, Japan) and photographed with a digital camera (c-5050, Olympus Corporation, Tokyo, Japan).

### Flow cytometry analysis

The expression of TLR8 in neutrophils was assessed by a FACS calibur flow cytometer (Becton Dickinson, San Jose, CA) according to the manufacturer's instructions. Briefly, 200 μl of the neutrophil suspension containing 2 × 10^6 ^neutrophils were first permeabilized by 1 × permeabilizing solution (Becton Dickinson, San Jose, California, USA) for 30 min on ice to stain not only cell surface TLR8 but also endosomal TLR8, and then incubated with 4 μl of PE-conjugated anti-TLR8 antibody solution or its isotype-control for 20 min at 4°C. After washing, the samples were fixed by 500 μl of 1% paraformaldehyde for 10 min. Binding of each antibody was detected using CellQuest analysis software on a FACS Calibur (Becton Dickinson, San Jose, California, USA). Specific binding of each antibody was expressed as relative fluorescence that was calculated by the ratio of the mean fluorescence intensity for TLR8 to the mean fluorescence intensity for the isotype control.

### TLR stimulation

Isolated neutrophils were stimulated in 24-well tissue culture plates with various concentrations of R848, a ligand for TLR 7/8, or R837, a ligand for TLR7, for 24 hr at 37°C in a humidified atmosphere of 5% CO_2_. Cells were pretreated with various concentrations of H_2_O_2 _for 30 min prior to the stimulation with R848 [24]. To investigate the effects of the inhibitors or a scavenger on the IL-8 release, cells were further pretreated with each agent prior to the treatment with H_2_O_2 _as follows: bafilomycin, an inhibitor of endosomal acidification, for 15 min; N-acethyl-L-cysteine was for 10 min; MG-132, a proteosome inhibitor, for 60 min; and dexamethasone for 30 min. Media were harvested at 24 hours after treatment with R848 for subsequent enzyme-linked immunosorbent assays (ELISA) to measure various cytokine levels. Similarly, cells were harvested at the same time for flow-cytometry analysis, or western blotting.

### Measurement of cytokines

IL-8 expression was measured by sandwich ELISA (R&D System Europe, Abingdon, UK) according to the manufacturer's instructions. The lower detection limit was 16 pg/ml. The levels of IL-1β, IL-6, IL-10, IL-12 and TNF-α were measured by a Human Inflammation Cytokine Beads array kit (Becton Dickinson, San Jose, California, USA) according to the manufacturer's instructions.

### Measurement of superoxide generation

Neutrophils were pre-incubated with or without 50 μM H_2_O_2_, and then stimulated with various concentrations of R848 for 1 hr at 37°C. Cells were harvested, washed twice and resuspended in 10% FCS in RPMI 1640 at a concentration of 1 × 10^6 ^cells/ml. One ml cell suspensions were cultured at 37°C with 3 μM DHR-123 for 5 min and then with 12-o-tetradecanoylphorbol 13-acetate for 30 min at 37°C. The cells were cooled on ice, centrifuged, and resuspended in PBS. Stained cells were assessed by a flow-cytometer (Becton Dickinson, San Jose, California, USA). The amount of superoxide generation was evaluated by the relative fluorescence intensity of DHR-123 compared with that of the control group.

### Chemotaxis assay

Neutrophils were pre-incubated with or without 50 μM H_2_O_2 _and then stimulated with various concentrations of R848 for 1 hr. Cells were harvested, washed twice and resuspended in 10% FCS in RPMI 1640 at a concentration of 2 × 10^6 ^cells/ml. Chemotaxis assays were performed on plastic chemotaxis chambers (pore size: 3 μm; Kurabou, Osaka, Japan) according to the manufacturer's instructions. Briefly, 250 μl of RPMI 1640 containing IL-8 (0.3 ng/ml) were placed into the bottom wells and 100 μl of the neutrophil suspension were added into the top wells. The chambers were then incubated in a tissue-culture incubator at 37°C for 1 hr. The numbers of neutrophils that transmigrated to the bottom wells were counted using a flow-cytometer (Becton Dickinson, San Jose, California, USA). Results are shown as the ratio of the migrated cell number of each group to that of the control group.

### Elastase assay

Elastase release from the neutrophils was measured by a human PMN elastase ELISA kit (Bender Medsystems, Vienna, Austria) according to the manufacturer's instructions.

### Phosflow analysis of phosphorylated NF-kB p65

1 × 10^6 ^neutrophils were incubated with or without 50 μM H_2_O_2 _and stimulated with various concentrations of R848 for 1 hr. The phosphorylated NF-kB p65 levels were measured by the BD phosflow method (Becton Dickinson, San Jose, CA) according to the manufacturer's instructions.

### Western blotting

After stimulation, the neutrophils were centrifuged at 400 × g for 10 seconds and incubated on ice for 30 min with cold Triton buffer (1% Triton X-100, 150 mM NaCl, 20 mM Tris-HCl, pH 7.4, 1 mM EDTA, 2 mM diisopropylfluorophosphate, 5 μg/ml pepstatin A and 1 mM phenylmethylsulfonylfluoride). Then, the cell lysates were centrifuged at 12,000 × g for 10 min, collected and stored at -80°C. Cell lysates were mixed with the same volume of 2 × SDS loading buffer and separated with 12.5% gradient polyacrylamide gel (DRC Co. Ltd., Tokyo, Japan). After electrophoresis, the proteins were transferred to a nitrocellulose membrane and incubated with anti-human MyD88 antibody (1:200 dilution), anti-human TRAF6 (1:200 dilution), or anti-human IkBα (1:200 dilution) overnight. To standardize the expression of each protein, the membranes were stripped off and re-probed with anti-β-actin antibody (1:10000 dilution). The membranes were then incubated with the appropriate peroxidase-conjugated secondary antibodies (1:2000 dilution). The bound antibodies were visualized with an ECL-plus detection system (Amersham, Backinghamshire, UK) and photographed by an ECL minicamera (Amersham, Backinghamshire, UK).

### Stastical analysis

Data are expressed as mean values ± SEM. Data were analyzed by one way analysis of variance (ANOVA) followed by Bonferroni's test or Sheffe's test to adjust for multiple comparisons. An unpaired two-tailed Student's t-test was used for single comparisons. Probability values of less than 0.05 were considered significant.

## Results

### Detection of toll-like receptor (TLR) 8 in human polymorphonuclear cells (PMNs) and its reaction to R848

**Figure 1:**
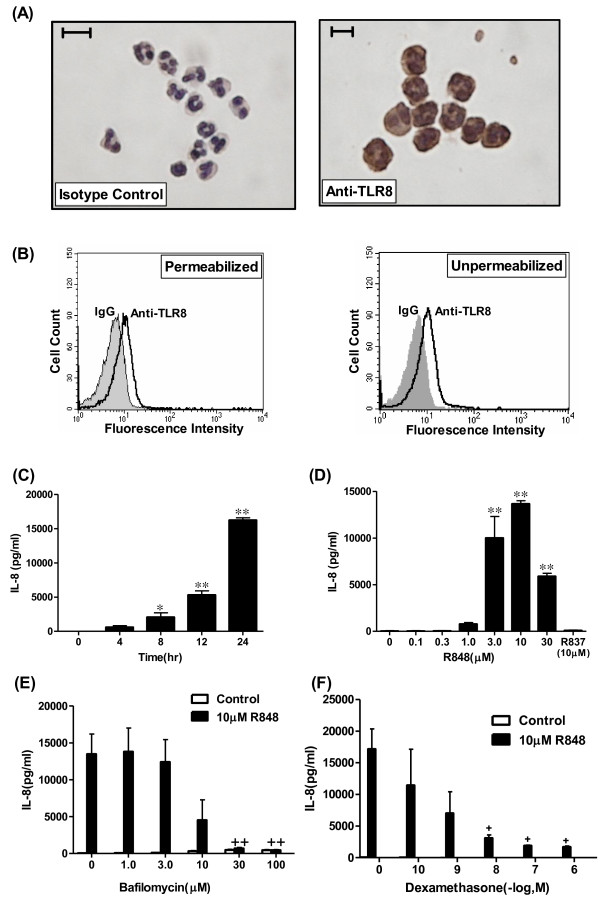
**Detection of toll-like receptor (TLR) 8 in human polymorphonuclear cells (PMNs), and the effects of TLR 7/8 ligand R848 on interleukin(IL)-8 release**. (A) TLR8 in PMN was detected by immunocytochemistry. Left panel indicates isotype control. Right panel shows TLR8 immunoreactivity in PMN. (Original magnification: × 400, Scale bars = 10 μm). (B) TLR8 expression was analyzed by flow-cytometry. PMNs were stained by anti-human TLR8 (solid lines) or the isotype control (gray histograms) in the permeabilized (left panel) and unpermeabilized condition (right panel). Left panel indicates both intercellular and cell surface expression of TLR8. Right panel shows cell surface expression alone. (C-F) Effect of R848 on the release of IL-8, and effect of bafilomycin or dexamethasone on the R848-induced IL-8 release from PMN. (C) PMNs were treated with 10 μM R848. The media were harvested at various time points and assayed for IL-8 by ELISA. (D) PMNs were treated for 24 hrs with R837, a ligand of TLR7, or various concentrations of R848, a ligand of TLR 7/8. Media were assayed for IL-8 by ELISA. (E, F) PMNs were treated with 10 μM R848 or vehicle in the presence of various concentrations of bafilomycin, an inhibitor of endosomal acidification (E), or dexamethasone (F). Media were assayed for IL-8 by ELISA. All values are mean values ± SEM of three to four separate experiments. *p < 0.05, **p < 0.01, compared with the values of control; +p < 0.05, ++p < 0.01, compared with the values of the vehicle-pretreated and 10 μM R848-treated group.

To determine whether human neutrophils express TLR8, we first investigated the expression of TLR8 in neutrophils by immunocytochemistry and flow-cytometry. As shown in Figure 1A, TLR8 was detected by immunocytochemistry. To examine the cellular localization of TLR8, we performed flow-cytometry analysis against TLR8. TLR8 was stained with or without cell membrane permeabilization, indicating that TLR8 exists not only in the cytosol such as the endosome but also on the cell surface (Figure 1B).

We next investigated the effect of TLR7 ligand R837 or TLR 7/8 ligand R848 on the release of IL-8 from neutrophils. R848 increased IL-8 release in a time-dependent manner (Figure 1C). As shown in figure 1D, R848 dose-dependently augmented the release of IL-8 at 24 hr, whereas R837 had no effect. To confirm whether this augmentation of IL-8 release is mediated by TLR signaling, the cells were pretreated with bafilomycin, an inhibitor of endosomal acidification. Pretreatment with bafilomycin significantly inhibited the R848-augmented IL-8 release in a dose-dependent manner (Figure 1E). Dexamethasone also significantly inhibited the R848-augmented IL-8 release (Figure 1F).

### Effect of H_2_O_2 _on R848-augmented cytokine release, superoxide generation, elastase release, and chemotaxis in human PMNs

**Figure 2:**
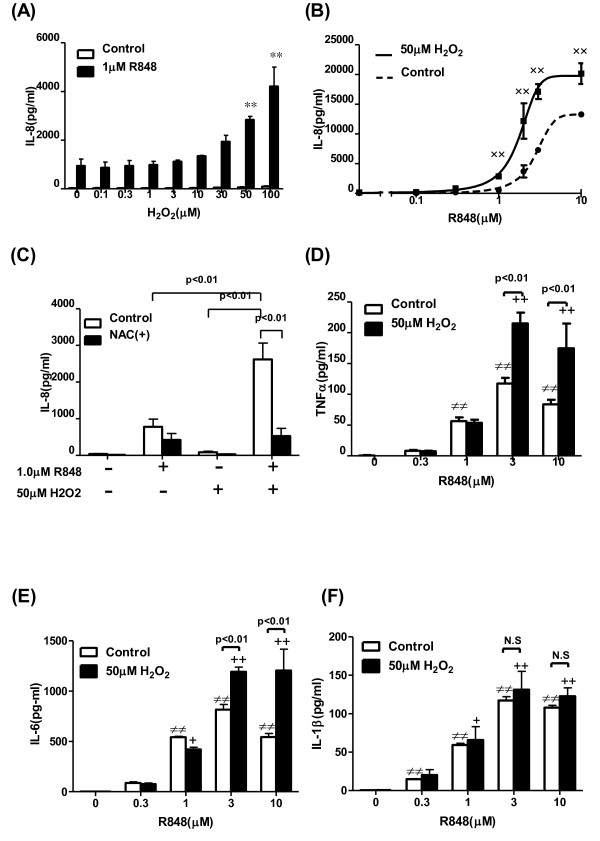
**Effect of H_2_O_2 _on the R848-induced cytokine release from human PMNs, and effect of N-acethyl-L-cysteine on the potentiation of cytokine release by H_2_O_2_**. (A) PMNs were incubated with various concentrations of H_2_O_2 _for 30 min, and then treated with R848 for 24 hrs. Media were assayed for IL-8 by ELISA. (B) Various concentrations of R848 were added to PMNs in the presence or absence of 50 μM H_2_O_2_. After 24 hrs, IL-8 levels in media were measured by ELISA. Dose-response curve of IL-8 release from PMNs was plotted against the R848 concentration. (C) Ten mM N-acethyl-L-cysteine (NAC) was added 10 min before H_2_O_2 _or vehicle treatment, then the PMNs were cultured for 24 hrs in the presence or absence of R848. (D-F) Effects of H_2_O_2 _on TNF-α (D), IL-6 (E) and IL-1β (F) release from the R848-treated PMNs were assessed by Cytokine-Beads Array. All values are mean values ± SEM of three to five separate experiments. **p < 0.01, compared with the values of vehicle-pretreated 1 μM R848-treated group; ^××^p < 0.01, compared with the values of control; ≠≠p < 0.01, compared with the values of vehicle treated group; +p < 0.05, ++p < 0.01, compared with the values of 50 μM H_2_O_2_-pretreated and vehicle-treated group.

**Figure 3:**
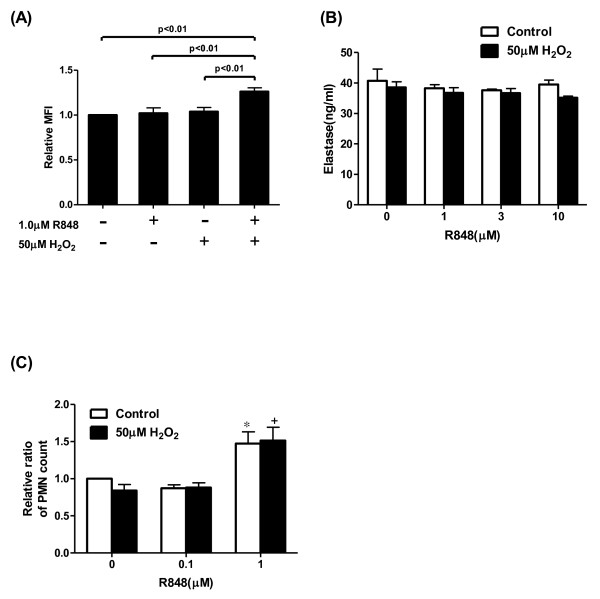
**Effect of H_2_O_2 _on the R848-induced superoxide generation, elastase release and chemotaxis in human PMNs**. (A) PMNs were preincubated for 30 min with or without 50 μM H_2_O_2_, and treated with vehicle or R848. Cells were then harvested and incubated with dihydro-rhodamine-123 (DHR-123) for 5 min. The amount of superoxide generation was indicated as the relative fluorescence intensity of DHR-123. (B) After incubation with or without 50 μM H_2_O_2_, PMNs were stimulated with various concentrations of R848 for 24 hrs. The media were assayed for elastase release by ELISA. (C) After one hour treatment with various concentrations of R848 with or without 50 μM H_2_O_2_, chemotactic capacity toward IL-8 was assessed by a modified boyden chamber method. Vertical axis: Relative ratio of the PMN counts (-fold increase). Relative ratio of the PMN counts was calculated as the ratio of the migrated cell count of each group to that of the control group. All values are mean values ± SEM of three to four separate experiments. *p < 0.05, compared with the values of vehicle-treated group; +p < 0.05, compared with the values of 50 μM H_2_O_2_-pretreated and vehicle-treated group; MFI = mean fluorescence intensity.

To examine whether oxidative stress potentiates the R848-augmented IL-8 release, we examined the effects of H_2_O_2 _on the IL-8 release from neutrophils. Pretreatment with H_2_O_2 _significantly potentiated the R848-augmented IL-8 release in a dose-dependent manner (Figure 2A). Pre-incubation with 50 μM H_2_O_2 _shifted the dose-response curve leftward (Log EC_50 _2.757 vs. 1.775 μM, p < 0.01, Figure 2B). In addition, the maximal response by R848 was also significantly potentiated compared with control (Figure 2B). This potentiation was abolished by an antioxidant, N-acetyl-L-cysteine, compared with the vehicle-pretreatment group (Figure 2C). The effect of R848 on the release of cytokines and the potentiation by H_2_O_2 _were also examined. As shown in Figure 2D–F, R848 significantly augmented TNF-α, IL-6 and IL-1β release from neutrophils. H_2_O_2 _potentiated the R848-augmented TNF-α (Figure 2D) and IL-6 release (Figure 2E) as well as IL-8, but H_2_O_2 _caused no potentiation of the IL-1β release (Figure 2F). Furthermore, we investigated whether H_2_O_2 _potentiated the R848-induced neutrophilic responses, including superoxide generation, elastase release, and chemotaxis. Neither H_2_O_2 _nor R848 stimulated superoxide production on their own, but the combination of the two did (Figure 3A), whereas H_2_O_2 _did not cause any potentiation of the elastase release and chemotactic capacity (Figure 3B and 3C).

### Effect of H_2_O_2 _on the R848-mediated TLR8 signaling

**Figure 4:**
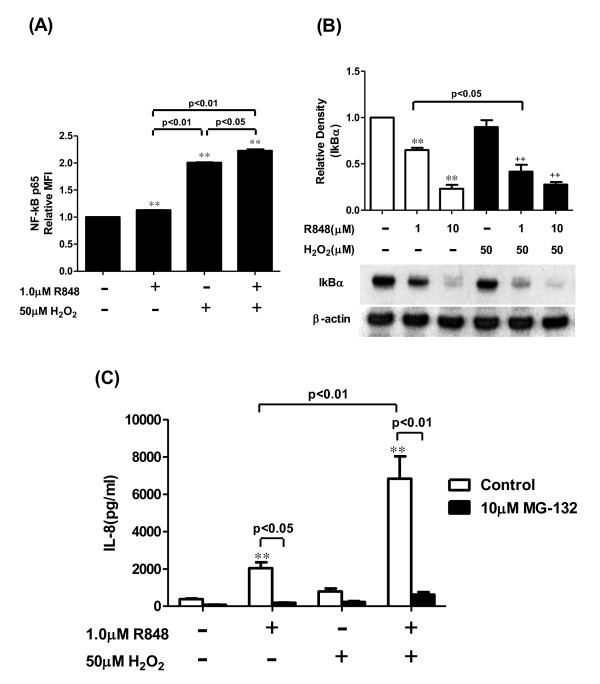
**Effect of H_2_O_2 _on the R848-induced nuclear factor-kappa B (NF-kB) activation**. Cells were treated with or without 50 μM H_2_O_2_, and then further treated with various concentrations of R848 for 60 min. Phosphorylated NF-kB p65 was assayed by a flow cytometer (A), and nuclear factor of kappa light polypeptide gene enhancer in B-cells inhibitor, alpha (IkBα) protein levels were assayed by western blotting (B). Each band intensity was assessed by densitometry. Relative intensity was calculated as the ratio of the specific band intensity to that of each appropriate β-actin band intensity. (C) PMNs were treated with 1 μM R848 with or without 50 μM H_2_O_2 _in the presence or absence of MG-132, a proteosome inhibitor. After 24 hrs, the media were assayed for IL-8 by ELISA. All values were mean values ± SEM of three to five separate experiments, and analyzed by ANOVA followed by Bonferroni's test. **p < 0.01, compared with the values of control; ++p < 0.01, compared with the values of H_2_O_2_-pretreated and vehicle-treated group; NF-kB p65 = nuclear factor-kappa B p65; IkBα = nuclear factor of kappa light polypeptide gene enhancer in B-cells inhibitor, alpha.

To clarify the mechanisms of the potentiation of the R848-induced neutrophilic responses by H_2_O_2_, we investigated whether H_2_O_2 _modulates the NF-kB activation induced by R848, which is a key signaling in TLR activation. Although R848 or H_2_O_2 _enhanced the phosphorylation of NF-kB p65, the phosphorylation was significantly augmented by the combination of R848 and H_2_O_2 _(Figure 4A). To investigate the mechanisms in the enhancement of NF-kB p65 phosphorylation by H_2_O_2_, we examined the effect of H_2_O_2 _on IkBα expression in the presence of R848. As shown in Figure 4B, R848 treatment dose-dependently reduced the IkBα protein levels. Furthermore, 50 μM H_2_O_2 _significantly reduced the IkBα protein level in the R848-treated cells, suggesting that H_2_O_2 _could modulate the NF-kB activity through the regulation of IkBα expression. Because NF-kB regulates IL-8 gene expression, we examined the effect of MG-132, a proteosome inhibitor, on the IL-8 release in the presence of R848 and H_2_O_2_. Pretreatment with MG-132 dose-dependently inhibited IkBα degradation as estimated by western blotting (Additional file 1). MG-132 also significantly reduced the augmented IL-8 release by treatment with R848 and H_2_O_2 _(Figure 4C). Furthermore, we evaluated whether H_2_O_2 _affected the amounts of TLR8, MyD88 and TRAF6, which are thought to be key molecules in TLR8 signaling. H_2_O_2 _did not affect these protein amounts in the presence of R848 (data not shown).

### Effect of dexamethasone on the H_2_O_2_-potentiated IL-8 release

**Figure 5:**
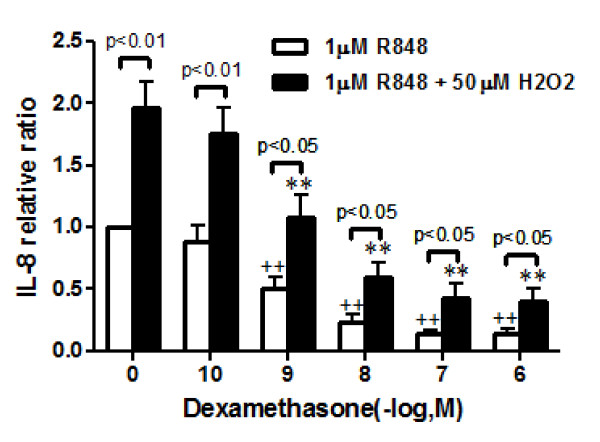
**Effect of dexamethasone on the H_2_O_2_-potentiated IL-8 release in the R848-treated PMNs**. PMNs were treated with or without dexamethasone for 30 min before treatment with or without 50 μM H_2_O_2_. Then, cells were treated with R848 for 24 hours. Media were assayed for IL-8 by ELISA. Vertical axis: IL-8 relative release (-fold increase). IL-8 relative ratio was calculated as follows: IL-8 relative ratio = IL-8 levels in the various conditions/IL-8 levels in the vehicle-pretreated and R848-treated condition. All values are mean values ± SEM of six separate experiments. ++p < 0.01, compared with the values of vehicle pretreated R848 treated group; **p < 0.01, compared to the values of H_2_O_2 _pretreated R848 treated group.

Because steroids have been used for viral infection-induced exacerbations of various pulmonary diseases such as bronchial asthma or COPD, we examined the effect of dexamethasone on the H_2_O_2_-potentiated IL-8 release in the R848 treated cells. As shown in Figure 5, dexamethasone dose-dependently reduced the H_2_O_2_-potentiated IL-8 release in the presence of R848. However, the inhibitory effects of dexamethasone were lower in the H_2_O_2 _and R848 combination treatment group than in the R848 treatment group.

## Discussion

In the current study, we have shown that peripheral blood neutrophils from healthy never-smoking subjects expressed TLR8, and that the TLR 7/8 ligand R848, but not the TLR7 ligand, induced IL-8 release from neutrophils. H_2_O_2 _potentiated the R848-augmented IL-8 release, and this potentiation was reversed by N-acetyl-L-cysteine. In addition, H_2_O_2 _potentiated the release of TNF-α and IL-6, and the superoxide generation in the R848 treated neutrophils. Although the expressions of TLR8, MyD88 and TRAF6 were not affected by H_2_O_2_, H_2_O_2 _enhanced the phosphorylation of NF-kB and potentiated the IkBα degradation in the R848 treated cells. Furthermore, MG-132, a proteosome inhibitor, reversed the H_2_O_2_-potentiated IL-8 release in the R848 treated neutrophils. These results suggested that oxidative stress potentiated the release of various R848-induced cytokines and superoxide generation in human neutrophils through NF-kB activation.

Previous reports have demonstrated that human peripheral blood neutrophils possessed all known TLRs except TLR3, but the expression levels of TLR7 and its reponses are extremely limited [16]. In the present study, R848, a potent synthetic agonist of TLR 7/8, but not the TLR7 ligand R837, enhanced the neutrophilic responses including the cytokine production (IL-8, TNF-α, IL-6 and IL-1β), the superoxide generation and the chemotaxis of neutrophils. This is consistent with a previous study, which showed that the influenza virus and R848 stimulated the IL-8 release in neutrophils through the activation of TLR 7/8 [21]. It was also shown that TLR7 knockout neutrophils respond poorly to both the TLR 7/8 ligand and the influenza virus in comparison with wild type neutrophils, suggesting that TLR7 plays an essential role in murine neutrophils. These results are inconsistent with our current study. However, several studies have reported that TLR7 stimulation affects the cytokine release not in human neutrophil, but in murine neutrophils [25,26]. These results suggest that the discrepancy of the findings with the previous report might be due to differences in the species.

In the current study, we showed that H_2_O_2 _potentiated the cytokine release including IL-8, TNF-α, and IL-6, and the superoxide generation in R848-treated neutrophils. In addition, this potentiation was reversed by N-acethyl-L-cysteine suggesting that oxidative stress is associated with the potentiation of the R848-mediated neutrophilic response. A previous report has shown that H_2_O_2 _pre-incubation potentiated lipopolysaccharide-induced IL-8 production, and that hydroxy radical scavengers markedly suppressed this potentiation [9,10,27]. These results are consistent with our findings. Although H_2_O_2 _potentiated the R848-augmented neutrophilic responses, the potentiation seemed to be heterogeneous. Indeed, H_2_O_2 _potentiated the R848-augmented IL-8, TNF-α, and IL-6 release, but did not potentiate the IL-1β release. This was an interesting finding because the degree of oxidative stress may modulate the profile of inflammatory mediators during viral infection. In the current study, it remained unclear why the potentiation by oxidative stress was heterogeneous. A future study is needed to explore this issue.

Hydrogen peroxide enhanced the R848-induced phosphorylation of NF-kB, and potentiated the degradation of IkBα. In addition, a proteosome inhibitor, MG-132, inhibited the H_2_O_2_-augmented IL-8 release in the R848-treated neutrophils. Considering that H_2_O_2 _did not affect the expression levels of TLR8 or other signaling molecules such as MyD88 or TRAF6, these results suggested that the H_2_O_2_-potentiated NF-kB activation could play a central role in the augmentation of the neutrophilic responses. This was consistent with previous reports, which have shown that oxidative stress cooperatively activated NF-kB with other mediators such as TNF-α [28-30].

In Figure 4A and 4B, the phosphorylation of NF-kB p65 in the vehicle-pretreated and R848-treated group was less than in the H_2_O_2_-pretreated and vehicle-treated group. In theory, the phosphorylation in the vehicle-pretreated and R848-treated group should be greater than in the H_2_O_2_-pretreated and vehicle-treated group. There is a possible explanation for this discrepancy. Generally, NF-kB is phosphorylated by NF-kB kinase and IkBα kinases when NF-kB is dissociated from IkBα and translocated into the nucleus in various types of cells [31,32]. There is no report that explored the interaction between NF-kB phosphorylation and IkBα degradation in neutrophils under TLR8 activation. Therefore, the finding observed in the current study may be due to an unknown signaling in the R848-treated neutrophils.

Steroids have been reported to reduce the severity and duration of admission in exacerbations of COPD and asthma. In this study, dexamethasone inhibited the R848-augmented IL-8 release from neutrophils in a dose-dependent manner, and this inhibition was observed in the presence or absence of H_2_O_2_. These results might indicate that steroids are useful therapeutic agents to attenuate the viral-induced neutrophilic inflammation. However, the pretreatment with H_2_O_2 _attenuated the effect of dexamethasone, suggesting that oxidative stress induced the steroid resistance. It has been reported that oxidative stress attenuates the effects of steroids in macrophages and epithelial cells through histone deacetylase 2 inactivation [24,33]. This mechanism may also explain the results observed in the present study.

There are several limitations in the current study. First, we used H_2_O_2 _as a model of oxidative stress. Many previous reports used this in vitro model to mimic the pathophysiological condition of oxidative stress observed in inflammatory lung diseases including COPD and asthma. We used H_2_O_2 _at 0.1 – 100 μM in the current study and these concentrations are the same range as in previous reports [24,34]. However, we should be careful when extrapolating the findings obtained in this in vitro model to the "real" pathophysiological conditions in inflammatory lung diseases. Second, we used neutrophils isolated from healthy subjects, not from smokers or patients with lung diseases. According to previous reports, the characteristics of neutrophils are altered in patients with COPD compared with healthy subjects [23,35]. The neutrophilic responses to TLR activation may be altered in patients with inflammatory lung disease. Third, we used R848 as a synthetic ligand for TLR 7/8. Many reports have used R848 as the ligand [16,21,22]. The stimulation of TLR 7/8 by R848 might be different from that of single strand RNA virus infection. In the current study, we attempted to elucidate the effects of oxidants on the TLR8 signaling. To accomplish this, we used R848 for the following reasons. First, R848 is a stable agent and is easy to handle compared with single strand RNA. Second, R848 does not have any other effect except TLR 7/8 stimulation. Indeed, the R848 signaling was abolished by treatment with bafilomycin, an inhibitor of endosomal acidification. Therefore, the findings in the current study seemed to be mediated by TLR8 signaling.

In conclusion, we have shown that the TLR8-mediated neutrophilic responses in healthy never-smoking subjects were markedly potentiated by oxidative stress, and this potentiation was mediated by enhanced NF-kB activation. These results suggested that oxidative stress might potentiate the neutrophilic inflammation during viral infection.

## Abbreviations

COPD: Chronic obstructive pulmonary disease; TLR8: Toll-like receptor 8; H_2_O_2_: Hydrogen peroxide; NF-kB p65: Nuclear factor-kappa B p65; IkBα: Nuclear factor of kappa light polypeptide gene enhancer in B-cells inhibitor, alpha; MyD88: Myeloid differentiation primary response gene 88; TRAF6: Tumor necrosis factor receptor-associated factor 6.

## Competing interests

The authors declare that they have no competing interests.

## Authors' contributions

SY carried out the data analysis and drafted the manuscript. AK, HS, and MI participated in the design of the original study, and contributed substantially to the manuscript. TI, MK, RT, KA, TH, KM and YM assisted with data analysis and interpretation, and supervised statistical analysis.

## Supplementary Material

Additional file 1 **Effect of MG-132 on the R848-induced nuclear factor of kappa light polypeptide gene enhancer in B-cells inhibitor, alpha (IkBα) degradation**. PMNs were incubated with or without 10 μM MG-132, a proteosome inhibitor, and then further treated with various concentrations of R848 for 60 min. The cytoplasmic fraction of cell lysates were used for estimating the protein levels of IKBα by western blotting. Each band intensity was assessed by densitometry. Relative intensity was calculated as the ratio of specific band intensity to that of each appropriate β-actin band intensity. All values are mean values ± SEM of three separate experiments. **p < 0.01; compared with the values of vehicle-treated group, IkBα = nuclear factor of kappa light polypeptide gene enhancer in B-cells inhibitor, alpha, n.s. = not significant. Click here for file
