# Supplementary figures and images for: Oxidative stress augments toll-like receptor 8 mediated neutrophilic responses in healthy subjects
Source: Respir Res. 2009 Jun 15;10(1):50. doi: 10.1186/1465-9921-10-50 (PMC2704194; doi:10.1186/1465-9921-10-50)

## Additional file 1.

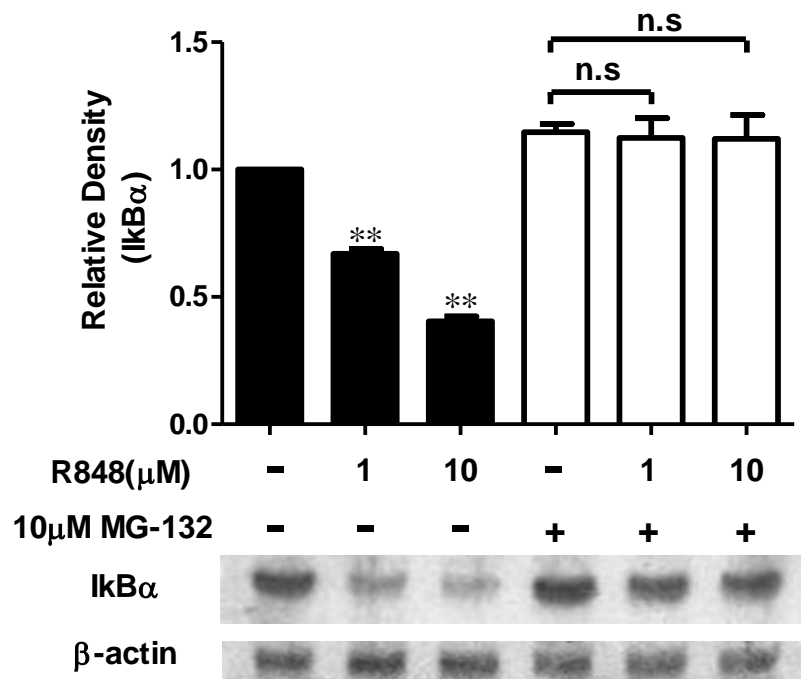

Supplement: Additional file 1 — Effect of MG-132 on the R848-induced nuclear factor of kappa light polypeptide gene enhancer in B-cells inhibitor, alpha (IkBα) degradation. PMNs were incubated with or without 10 μM MG-132, a proteosome inhibitor, and then further treated with various concentrations of R848 for 60 min. The cytoplasmic fraction of cell lysates were used for estimating the protein levels of IKBα by western blotting. Each band intensity was assessed by densitometry. Relative intensity was calculated as the ratio of specific band intensity to that of each appropriate β-actin band intensity. All values are mean values ± SEM of three separate experiments. **p < 0.01; compared with the values of vehicle-treated group, IkBα = nuclear factor of kappa light polypeptide gene enhancer in B-cells inhibitor, alpha, n.s. = not significant. [file 1465-9921-10-50-S1.pdf]
